# Model for individual prediction of diabetes up to 5 years after gestational diabetes mellitus

**DOI:** 10.1186/s40064-016-1953-7

**Published:** 2016-03-11

**Authors:** Claes Ignell, Magnus Ekelund, Eva Anderberg, Kerstin Berntorp

**Affiliations:** Department of Clinical Sciences, Malmö, Lund University, Malmö, Sweden; Department of Obstetrics and Gynecology, Helsingborg Hospital, SE-251 87 Helsingborg, Sweden; Department of Internal Medicine, Helsingborg Hospital, Helsingborg, Sweden; Department of Clinical Sciences, Lund, Lund University, Lund, Sweden; Department of Endocrinology, Skåne University Hospital, Malmö, Sweden

**Keywords:** Diabetes mellitus, Follow-up, Gestational diabetes mellitus, Glucose tolerance, Prediction

## Abstract

**Aims:**

To identify predictors of diabetes development up to 5 years after gestational diabetes mellitus (GDM) and to develop a prediction model for individual use.

**Methods:**

Five years after GDM, a 75-g oral glucose tolerance test (OGTT) was performed in 362 women, excluding women already diagnosed with diabetes at 1- to 2-year follow-up or later (*n* = 45). All but 21 women had results from follow-up at 1–2 years, while 84 women were lost from that point. Predictive variables were identified by logistic regression analysis.

**Results:**

Five years after GDM, 28/362 women (8 %) were diagnosed with diabetes whereas 187/362 (52 %) had normal glucose tolerance (NGT). Of the latter, 139/187 (74 %) also had NGT at 1- to 2-year follow-up. In simple regression analysis, using NGT at 1–2 years and at 5 years as the reference, diabetes at 1- to 2-year follow-up or later was clearly associated with easily assessable clinical variables, such as BMI at 1- to 2-year follow-up, 2-h OGTT glucose concentration during pregnancy, and non-European origin (*P* < 0.0001). A prediction model based on these variables resulting in 86 % correct classifications, with an area under the receiver-operating characteristic curve of 0.91 (95 % CI 0.86–0.95), was applied in a function-sheet line diagram illustrating the individual effect of weight on diabetes risk.

**Conclusions:**

The results highlight the importance of BMI as a potentially modifiable risk factor for diabetes after GDM. Our proposed prediction model performed well, and should encourage validation in other populations in future studies.

## Background

The prevalence of gestational diabetes mellitus (GDM) is increasing worldwide. Currently, it affects about 2.6 % of pregnant women in southern Sweden (Hunt and Schuller 2007; Ignell et al. 2014). GDM is an important risk factor for type-2 diabetes and cardiovascular disease (Bellamy et al. 2009; Harreiter et al. 2014). A cumulative diabetes incidence of 30–50 % within 5–10 years after GDM has been described (Kim et al. 2002; Ekelund et al. 2010). GDM and type-2 diabetes have many risk factors in common (Dornhorst and Rossi 1998), and both are characterized by insulin resistance and an inability of the beta cells to compensate by a sufficient increase in insulin secretion (Buchanan et al. 2007; Retnakaran et al. 2008). However, the incidence of GDM and type-2 diabetes following GDM is dependent of the screening activity and the diagnostic criteria used to define GDM. In southern Sweden, universal screening with a 75-g oral glucose tolerance test (OGTT) has been used since 1995. Using the World Health Organization (WHO) criteria from 1999 to define GDM, we have previously reported a diabetes incidence of 6 % 1–2 years after delivery (Anderberg et al. 2011; World Health Organization 1999). Here we report the results of 5-year follow-up of these women.

As intervention studies have shown that type-2 diabetes can be prevented by modification of lifestyle (Knowler et al. 2002; Lindstrom et al. 2003), even in women with a history of GDM (Ratner et al. 2008; Aroda et al. 2015), a major challenge in public healthcare is to identify individuals who have the highest risk (Noble et al. 2011). The aim of the present study was to identify risk factors associated with diabetes development up to 5 years after pregnancy and to explore the possibility of establishing a model for diabetes prediction that could be used in clinical practice on an individual basis when counseling women after GDM.

## Methods

### Study population

The present study was part of the Mamma Study, which was conducted in four of the five delivery departments in the county of Skåne in southern Sweden. The design of the study has been described in detail elsewhere (Anderberg et al. 2011; Ignell et al. 2013). Briefly, during the years 2003–2005, all pregnant women, representing different glucose categories according to an OGTT, were invited to take part in a 5-year follow-up program postpartum. A 75-g OGTT was offered to all women in the 28th week of gestation, and also in gestational week 12 if they had had a history of GDM in previous pregnancies or a first-degree relative with diabetes. The diagnostic criteria for GDM used in the present study were a modification of those recommended by the WHO in 1999, defining GDM as the joint category of diabetes and impaired glucose tolerance (IGT) based on the 2-h capillary plasma glucose concentration (World Health Organization 1999). A 2-h capillary plasma glucose concentration below the limit for IGT was considered to be normal glucose tolerance (NGT) during pregnancy (GNGT) (World Health Organization 1999).

The first follow-up appointment took place 1–2 years after delivery (Anderberg et al. 2011; Ignell et al. 2013). A 75-g OGTT was performed after overnight fasting in 470 women with previous GDM, and in 166 women with previous GNGT. Fasting and 2-h venous blood samples were drawn in duplicate for determination of plasma glucose concentration, and the mean value was calculated. Diagnostic criteria were those proposed by the WHO (World Health Organization 1999). Weight and height were measured and body mass index (BMI) calculated (kg/m^2^). Information on first-grade family history of diabetes, earlier pregnancies, smoking/snuff habits, and ethnic affiliation was obtained. Based on the stated country of origin of at least three grandparents, women were grouped according to whether they were of European or non-European origin. Using this definition, 14 women with previous GDM were unclassifiable. In all, 32 women were diagnosed with diabetes 1–2 years after delivery (all GDM) and were referred to primary care for clinical surveillance. During the two subsequent years, 13 other women with previous GDM (3 NGT and 10 IGT at 1–2 years) were given a diabetes diagnosis.

**Fig. 1:**
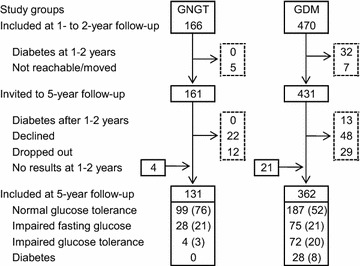
Flow chart of the study population and diagnoses at 5-year follow-up. Number of participants and *n* (%) for diagnosis at 5-year follow-up are given. *GNGT* gestational normal glucose tolerance, *GDM* gestational diabetes mellitus

The second and final follow-up took place 5 years after pregnancy and followed the same procedure as the 1- to 2-year follow-up, except for capillary blood sampling during the OGTT for the determination of plasma glucose concentrations. A flow chart of the study population is shown in Fig. 1. In addition to the 45 women already diagnosed with diabetes after GDM, 84 women with GDM and 29 women with GNGT were lost to follow-up at this point. However, 25 women who did not attend the 1- to 2-year appointment attended the 5-year appointment (4 GNGT, 21 GDM).

Informed consent was obtained from all individual participants included in the study, and the study protocol was approved by the Ethics Committee of Lund University, Sweden (LU 259-00).

### Metabolic measurements

The HemoCue Glucose 201+ system (HemoCue AB, Ängelholm, Sweden) was used for immediate measurement of plasma glucose concentrations (mmol/L). The mean coefficient of variation of the duplicate capillary analyses performed in this study was 2.4 %, and that for the venous analyses was 2.6 %.

### Statistical analysis

Data are presented as *n* (%) for categorical variables and as median (interquartile range) for continuous variables. Fisher’s exact test was used to compare group frequencies and the Mann–Whitney U test was used to compare group differences between medians. Simple logistic regression analysis was used to calculate Nagelkerke R^2^, and odds ratios (ORs) with 95 % confidence interval (CI). Variables tested for associations with diabetes after GDM were non-European origin (yes/no), first-grade diabetes heredity (yes/no), age at delivery (years), glucose concentrations during OGTT (mmol/L), time interval to follow-up (years), BMI at follow-up (kg/m^2^), and parity, which was best expressed as up to three deliveries at follow-up versus more than three (≤3/>3). Diagnosis in early gestation (yes/no) and insulin treatment during pregnancy (yes/no) were also analyzed, but they were not included in the final multiple model since these variables were deemed less stable depending on the screening strategy and the judgement by the physician.

Multivariable logistic regression analysis was done with either backward elimination of non-significant factors or forward adding of significant factors. Probability of diabetes (%) in the prediction model was calculated from the function: *F*(*t*) = *e*^*t*^/(1 + *e*^*t*^), where *t* is represented by the equation from the final multivariable regression (Hosmer and Lemeshow 2005). The performance of the prediction model was assessed in receiver-operating characteristic (ROC) curves with calculations of area under the curve (AUC). Threshold for discrimination was calculated with the Youden index (Hajian-Tilaki 2013).

IBM SPSS Statistics 22 for Windows (IBM Corporation, Armonk, NY) was used for analysis. Two-sided *P* values of less than 0.05 were considered to be statistically significant.

## Results

Altogether, 131 women with GNGT and 362 women with GDM had an OGTT 5 years postpartum (Fig. 1). Frequencies of overweight (BMI ≥ 25 kg/m^2^) in these groups were 42 and 44 %, respectively, at the 1- to 2-year follow-up (*P* = 0.50), and 47 % in both groups at the 5-year follow-up. None of the women with GNGT were diagnosed with diabetes at the 1- to 2-year follow-up or later, whereas in addition to the 45 women already diagnosed with diabetes, 28 other women with previous GDM were diagnosed with diabetes at the 5-year appointment. Of the 72 women with IGT 5 years after GDM, 32 (44 %) also had impaired fasting glucose (IFG).

Of the 362 women with previous GDM, 341 also had results from the 1- to 2-year follow-up. Adding the 45 women already diagnosed with diabetes at 1- to 2-years or later, altogether 72/386 (19 %) of the women had a diabetes diagnosis 5 years after GDM. In women with IFG or IGT at the 1- to 2-year OGTT, 18/117 (15 %) had diabetes at the 5-year OGTT. The corresponding figure in women with NGT at the 1- to 2-year OGTT was 9/224 (4 %). Using NGT as a reference, IFG or IGT at 1- to 2-year follow-up was associated with an increased risk of diabetes up to 5 years postpartum (OR 5.1, 95 % CI 2.5–10.4, *P* < 10^−5^).

Comparing the 341 women who attended both the 1- to 2-year follow-up and the 5-year follow-up with the 84 women (without a previous diabetes diagnosis) who were lost to 5-year follow-up, there were no significant differences in clinical characteristics such as ethnic origin, first-grade diabetes heredity, age at delivery, 2-h glucose level during pregnancy, BMI or glucose levels during the OGTT at the 1- to 2-year follow-up.

In Table 1, clinical data from pregnancy and follow-up are given in relation to glucose category at the 5-year OGTT for women with previous GDM. Using NGT as a reference, women with diabetes were characterized by an increased frequency of non-European origin, higher 2-h glucose level during pregnancy, higher BMI at both follow-up visits, and higher fasting and 2-h glucose levels during the OGTT 1–2 years postpartum. Similarly, women with IFG/IGT had higher BMI than women with NGT. Snuff was used in less than 1 % of the women during pregnancy and follow-up, whereas 5 % smoked during pregnancy (as compared to 9–10 % during follow-up). There were no significant differences in the frequencies of tobacco use during pregnancy or follow-up between women with GNGT and women with GDM; nor were there any differences in the frequencies of smoking related to glucose tolerance at 5-year follow-up.

**Table 1:** Descriptive data from pregnancy and follow-up in relation to glucose category 5 years after GDM

	NGT (*n* = 187)	IFG (*n* = 75)	P*	IGT (*n* = 72)	P*	Diabetes (*n* = 28)	P*
Non-European origin	23 (13)	10 (15)	0.84	12 (18)	0.42	13 (48)	<0.001
First-grade diabetes heredity	47 (28)	24 (36)	0.27	28 (44)	0.027	16 (62)	0.001
Age at delivery (years)	32.1 (29.1–36.0)	32.3 (28.8–35.9)	0.99	34.6 (32.2–36.8)	0.002	35.4 (28.8–38.2)	0.060
*Pregnancy*							
2-h PG (mmol/L)	9.3 (8.9–9.9)	9.3 (8.9–10.3)	0.43	9.5 (9.1–10.3)	0.083	10.1 (9.6–10.8)	<0.001
Diagnosis in early gestation	8 (5.0)	5 (7.9)	0.53	7 (12)	0.13	6 (23)	0.006
Insulin treatment	12 (6.5)	7 (9.3)	0.44	8 (11)	0.20	6 (21)	0.018
*1*–*2* *years after pregnancy*							
Interval to follow-up (years)	1.3 (1.0–1.7)	1.2 (1.0–1.5)	0.15	1.4 (1.2–1.6)	0.12	1.4 (1.1–1.7)	0.68
Deliveries >3	7 (4.0)	6 (8.5)	0.20	5 (7.2)	0.33	3 (10.7)	0.14
BMI (kg/m^2^)	23.0 (21.2–26.0)	24.1 (21.6–27.5)	0.010	24.4 (22.2–27.1)	0.011	27.0 (25.1–31)	<10^−6^
FPG (mmol/L)	5.2 (4.9–5.6)	5.6 (5.2–6.0)	<10^−4^	5.3 (5.0–5.8)	0.12	5.7 (5.2–6.3)	<0.001
2-h PG (mmol/L)	5.8 (5.0–6.9)	6.4 (5.5–7.0)	0.043	7.4 (6.1–8.3)	<10^−8^	7.2 (6.9–8.5)	<10^−6^
*5* *years after pregnancy*							
Interval to follow-up (years)	5.1 (5.0–5.2)	5.1 (5.0–5.3)	0.95	5.1 (5.0–5.3)	0.39	5.2 (5.0–5.5)	0.28
Deliveries >3	13 (7.0)	9 (12.0)	0.22	10 (13.9)	0.09	5 (18)	0.066
BMI (kg/m^2^)	23.4 (21.3–26.8)	25.0 (22.7–27.9)	0.005	25.8 (23.1–27.9)	0.001	28.0 (26.8–34.6)	<10^−7^
FPG (mmol/L)	5.5 (5.3–5.8)	6.3 (6.2–6.5)	<10^−35^	6.0 (5.6–6.4)	<10^−10^	7.1 (6.8–7.2)	<10^−15^
2-h PG (mmol/L)	7.4 (6.6–8.0)	7.7 (7.3–8.2)	0.003	9.7 (9.2–10.3)	<10^−34^	12.2 (9.5–12.7)	<10^−13^

Data given are *n* (%) or median (interquartile range)

Differences were tested with Fisher’s exact test (categorical variables) or the Mann–Whitney U test (continuous variables)

*GDM* gestational diabetes mellitus, *NGT* normal glucose tolerance, *IFG* impaired fasting glucose, *IGT* impaired glucose tolerance, *PG* plasma glucose, *FPG* fasting plasma glucose

* All comparisons were performed against NGT

To investigate which variables were associated with development of diabetes up to 5 years after GDM, women with NGT at 1- to 2-year follow-up and 5-year follow-up were used as reference (Table 2A). Of the variables tested for an association with diabetes in the multivariable analysis, ethnic origin, 2-h glucose concentrations during pregnancy, and BMI at 1- to 2-year follow-up remained after backward elimination, while age at delivery and first-grade diabetes heredity were not significant in this study. Change in BMI from 1- to 2-year follow-up to 5-year follow-up was not significantly associated with diabetes in multivariable analysis when related to BMI at the respective follow-up. One woman with a weight loss of 43 % after pregnancy due to bariatric surgery and NGT at 5-year follow-up was excluded in later analyses.

**Table 2:** Descriptive data and results of regression analyses for associations with diabetes 5 years after GDM

	A. After pregnancy						B. After IFG or IGT at 1–2 years		
	NGT at 1–2 years and 5 years (*n* = 139)	Diabetes at 1–2 years or later (*n* = 73)	P*	Simple regression			Simple regression^a^		
				R^2^	OR (95 % CI)	*P*	R^2^	OR (95 % CI)	*P*
Non-European origin	17 (13)	34 (51)	<10^−7^	0.21	7.09 (3.52–14.26)	<***10*** ^***−7***^	0.08	3.24 (1.02–10.31)	0.047
First-grade diabetes heredity	36 (27)	35 (54)	<0.001	0.09	3.14 (1.69–5.84)	<0.001	0.10	3.17 (1.11–9.05)	0.031
Age at delivery (years)	31.9 (29.1–36.0)	35.4 (30.5–38.2)	0.004	0.06	1.10 (1.03–1.17)	0.005	0.12	1.15 (1.02–1.30)	***0.022***
*Pregnancy*									
2-h PG (mmol/L)	9.3 (8.9–10.0)	10.1 (9.7–10.9)	<10^−5^	0.16	1.91 (1.41–2.58)	<***10*** ^***−4***^	0.35	4.32 (1.78–10.51)	***0.001***
Diagnosis in early gestation	8 (6.3)	16 (26)	<0.001	0.10	5.24 (2.10–13.10)	<0.001	0.19	–	1
Insulin treatment	7 (5.0)	21 (30)	<10^−5^	0.15	8.25 (3.30–20.64)	<10^−5^	0.17	6.00 (1.67–21.59)	0.006
*1‒2* *years after pregnancy* ^a^									
Interval to follow-up (years)	1.3 (1.0–1.7)	1.4 (1.1–1.8)	0.31	<0.01	1.32 (0.80–2.20)	0.28	0.03	0.54 (0.19–1.57)	0.26
Deliveries >3	5 (3.6)	11 (16)	0.004	0.06	5.08 (1.69–15.29)	0.004	0.13	10.00 (1.12–88.91)	0.039
BMI (kg/m^2^)	22.4 (20.8–24.7)	30.3 (25.8–35.4)	<10^−15^	0.40	1.28 (1.19–1.37)	<***10*** ^***−10***^	0.29	1.25 (1.10–1.41)	***0.001***
FPG (mmol/L)	5.2 (4.9–5.5)	6.2 (5.5–6.8)	<10^−16^		NA		0.12	3.2 (1.20–8.2)	0.019
2-h PG (mmol/L)	5.6 (4.8–6.3)	8.6 (7.0–11.2)	<10^−20^		NA		0.012	1.13 (0.82–1.5)	0.45
*5* *years after pregnancy* ^a^									
Interval to follow-up (years)	5.1 (5.0–5.2)	5.1 (5.0–5.3)	0.64	0.01	0.58 (0.25–1.34)	0.20	0.05	0.41 (0.11–1.50)	0.18
Deliveries >3	6 (4.3)	7 (20)	0.005	0.07	5.54 (1.73–17.75)	0.004	0.04	2.50 (0.59–10.61)	0.21
BMI (kg/m^2^)	22.8 (20.9–25.9)	28.0 (26.7–35.1)	<10^−9^	0.35	1.30 (1.18–1.43)	<10^−6^	0.31	1.30 (1.10–1.53)	0.002
FPG (mmol/L)	5.5 (5.2–5.8)	7.1 (6.7–7.2)	<10^−16^		NA			NA	
2-h PG (mmol/L)	7.3 (6.6–8.1)	12.1 (9.3–12.6)	<10^−11^		NA			NA	

Data given are *n* (%) or median (interquartile range)

Differences were tested with Fisher’s exact test (categorical variables) or the Mann–Whitney U test (continuous variables)

Variables used in prediction models A and B are marked with bolditalics *P* values

*GDM* gestational diabetes mellitus, *NGT* normal glucose tolerance, *IFG* impaired fasting glucose, *IGT* impaired glucose tolerance, *PG* plasma glucose, *FPG* fasting plasma glucose, *R* ^*2*^ R-squared by Nagelkerke, *OR* odds ratio, *NA* not applicable

* Comparisons performed against NGT at 1–2 years and 5 years

^a^Simple regression for diabetes after 1–2 years (*n* = 28) versus NGT at 5 years (*n* = 36)

Variables remaining after backward elimination in the multivariable regression analysis were used when constructing a model for diabetes prediction after GDM, including results from 200 women (67 diabetes, 133 NGT). Accordingly, ethnic origin (0 coding for European, and 1 coding for non-European, “E”), 2-h glucose concentration during pregnancy (“GP”), and BMI from the 1- to 2-year follow-up were used to generate model A for prognostication of diabetes risk (%) with NGT at 1–2 years and 5-years as reference: $\left( {{\text{Exp}}\left( {1.919*{\text{E}} + 0.703*{\text{GP}} + 0.274*{\text{BMI}}{-}15.5} \right)} \right)/\left( {1 + {\text{Exp}}\left( {1.919*{\text{E}} + 0.703*{\text{GP}} + 0.274*{\text{BMI}}{-}15.5} \right)} \right)*100$. In this population, model A correctly prognosticated 86 % of the women with diabetes after GDM, with an AUC of 0.91 (95 % CI 0.86–0.95). A calculated optimal cutoff for diabetes risk of 36.4 % yielded a sensitivity of 82.1 %, a specificity of 88.0 %, a positive predictive value of 77.5 %, and a negative predictive value of 90.7 %.

**Fig. 2:**
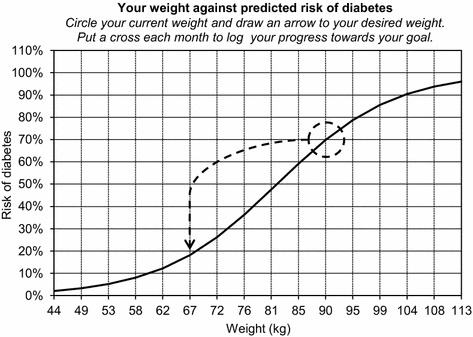
Line diagram representing an individually predicted risk of diabetes 5 years after GDM versus weight. This example illustrates the risk for a European woman with a height of 1.64 m, a 2-h OGTT capillary plasma glucose concentration of 10.2 mmol/L in pregnancy, and a current weight of 90 kg—resulting in a predicted 70 % risk of diabetes with a constant weight and declining to a 18 % risk with a weight loss of 23 kg

With the idea of using analyzed follow-up data with the purpose of individual counseling of women after pregnancy with GDM, we designed a function sheet with a line diagram relating possible weight to prognosticated risk of diabetes from model A. An individual example is shown in Fig. 2.

To investigate determinants of diabetes or NGT 5 years after GDM in women classified as IFG or IGT at 1- to 2-year follow-up, regression analyses were performed, adding significant variables in a forward strategy—as the quantity of women in this analysis was limited (Table 2B). Based on these findings, a prognostication model B was developed, resulting in 88 % correct classifications in the 64 women included (28 diabetes, 36 NGT), with an AUC of 0.93 (95 % CI 0.86‒0.99). An optimal cutoff of 54.9 % gave a sensitivity of 82.1 %, a specificity of 97.2 %, a positive predictive value of 95.8 %, and a negative predictive value of 87.5 %. The prognostication of diabetes risk (%) with NGT as reference was calculated as: $({\text{Exp}} \left( {0.215*{\text{AD}} + 2.156*{\text{GP}} + 0.271*{\text{BMI}}{-}35.783} \right)/\left( {1 + {\text{Exp}} \left( {0.215*{\text{AD}} + 2.156*{\text{GP}} + 0.271*{\text{BMI}}{-}35.783} \right)} \right)*100$, with “AD” representing age at delivery and “GP” representing 2-h plasma glucose concentration during pregnancy, and using BMI from the 1- to 2-year follow-up.

## Discussion

To the best of our knowledge, this is the first study to use long-term follow-up data after GDM in prediction models to aid in individual counseling. The prediction models performed well with 86 % correct classifications in model A and an AUC of 91 %. Prognostication for women with IFG or IGT 1–2 years after GDM in model B gave even better results, although these were based on fewer women. Excluding women previously diagnosed with diabetes, an additional 8 % of the women with previous GDM were diagnosed with diabetes at the 5-year follow-up, whereas none of the women with normal glucose tolerance during pregnancy were diagnosed with diabetes during the follow-up period. These findings suggest that the 2-h glucose threshold value of the WHO 1999 diagnostic criteria for GDM could be regarded as a watershed for diabetes development after GDM. However, the groups were not completely comparable, with high-risk ethnic origin in particular being overrepresented in women with GDM (Anderberg et al. 2011).

The variables included in the proposed models might well be accompanied or replaced by other risk factors in repeated studies and other populations. Age, first-grade diabetes heredity, and parity >3 were less stable predictors, which might be attributed to significant confounding with a non-European background in this population. Non-European origin was more frequently represented among women diagnosed with diabetes 5 years after GDM, as also previously described and analyzed from the 1- to 2-year follow-up (Ignell et al. 2013). In contrast, an Austrian group assessing a cluster of risk factors for diabetes manifestation up to 10 years after GDM did not observe any effect of non-European origin (Göbl et al. 2011). This discrepancy may be explained by differences in the composition of the non-European groups in the two studies. When using broader ethnic classifications, caution is warranted, as considerable differences can exist even within apparently well-defined populations (WHO Expert Consultation 2004).

When analyzing diabetes prediction up to 5 years after GDM, data from women with NGT at 1- to 2-year follow-up and 5-year follow-up were used as reference, to get a clear-cut discrimination (prediction model A). Furthermore, we found it worthwhile to investigate determinants of diabetes after being diagnosed with IFG or IGT at the 1- to 2-year follow-up, using NGT at 5 years as reference (prediction model B). However, the diagnosis of these intermediate glucose categories is known to be more prone to error than the diagnosis of diabetes, and there is an ongoing debate on how well these categories predict diabetes (Yudkin and Montori 2014). For this reason, and since model A was based on a larger number of women, we focused on model A—which was described in greater detail and used in the prediction model line diagram (Fig. 2). The cutoff points identified concerning prediction of diabetes risk, resulting in high predictive values for both models, may not be good enough to be used by clinicians to refrain from further follow-up. For this purpose, completing the models with more sophisticated analyses might prove to be effective (Barden et al. 2013).

A limitation of the study was the rather low overall participation rate in the Mamma Study; less than 50 % of consenting women with previous GDM took part in the 1- to 2-year follow-up (Anderberg et al. 2011). Studies have repeatedly shown poor compliance with recommended guidelines in clinical practice, and women fail to attend the postpartum visit, even in the research setting (Carson et al. 2013). As previously reported, the only information available concerning non-participants at the first follow-up was their age (Anderberg et al. 2011). This is a clear limitation since differences in known risk factors for diabetes, such as BMI, family history of diabetes and ethnic background between participants and non-participants will have an impact on the prevalence of diabetes after GDM. Nevertheless, 85 % of eligible women from the first follow-up took part in the 5-year follow-up, and it is a strength that their previously recorded data from the 1- to 2-year follow-up was not significantly different from the data from those who declined participation or dropped out. The participation rate at the first-follow-up might have been improved if follow-up had been performed at the regular maternal care visit 3 months after delivery, which would also have been valuable since early conversion to type-2 diabetes is not uncommon (Kim et al. 2002; Kwak et al. 2013; Capula et al. 2014). Unfortunately, we did not have access to weight in early or late gestation, fasting glucose concentrations at diagnosis in pregnancy, or HbA1c levels for the entire population—factors that could have improved the prediction models further (Capula et al. 2014; Kwak et al. 2013; Ekelund et al. 2010). Although BMI shows a good correlation with percentage of body fat, there are variations according to ethnicity and age (WHO Expert Consultation 2004). Measurements of waist circumference might also have added new and valuable data, especially if combined with height, as reported by Gruson et al. (WHO Expert Consultation 2004; Gruson et al. 2010; Wang et al. 2014).

Different models for prediction of type-2 diabetes in women with GDM have been presented recently (Cormier et al. 2015; Capula et al. 2014; Barden et al. 2013). Cormier et al. evaluated the predictive value in ROC curves of a genetic risk score with 36 single-nucleotide polymorphisms (Cormier et al. 2015). Combined with BMI and age, an AUC of 0.67 for the prediction of diabetes versus NGT was reported, based on 214 women with a mean follow-up time of 3.5 years after GDM. Including information on glucose concentration from pregnancy in prediction models appears to be of major importance. Capula et al. reported an AUC of 0.90 for fasting plasma glucose concentration at GDM screening for the prediction of diabetes 6–12 weeks postpartum (Capula et al. 2014). Furthermore, Barden et al. reported that a fasting plasma glucose concentration of >5.5 mmol/L at diagnosis during pregnancy was almost as effective as a “high-risk” cluster of cardiometabolic risk factors to predict diabetes 10 years after GDM (Barden et al. 2013). Similarly, in a previous study we found that a fasting blood glucose level of ≥5.2 mmol/L was associated with a fourfold to sixfold increased risk of diabetes 5 years after GDM (Ekelund et al. 2010).

The method of motivational interviewing has been shown to be useful when counseling to enhance weight loss (Armstrong et al. 2011). The concept of using a prediction model in a function-sheet line diagram to illustrate an individualized risk in relation to a modifiable risk factor might prove to be a useful tool for visual information and interaction when motivating women to a healthy lifestyle and to increase compliance to follow-up.

## Conclusion

The diagnostic 2-h OGTT glucose concentration during pregnancy, non-European origin, and BMI at the first postpartum follow-up was found to be associated with diabetes development up to 5 years after GDM. The results highlight the importance of BMI as a potentially modifiable risk factor for diabetes. Our proposed prediction models performed well with large proportions of correct classifications, and should encourage further validation in other populations in future studies.
